# Efficacy and safety of blonanserin versus risperidone in the treatment of schizophrenia: a systematic review and meta-analysis of randomized controlled trials

**DOI:** 10.1186/s12888-023-05240-7

**Published:** 2023-10-11

**Authors:** Shu-Wen Deng, Qian Xu, Wen-Long Jiang, Bo Hong, Bo-Hui Li, Da-Wei Sun, Hai-Bo Yang

**Affiliations:** 1 College of Pharmacy, Qiqihar Medical University, Qiqihar, China; 2 The 8th Affiliated Hospital of Qiqihar Medical University, No.192 Rangdu Road, Ranghulu district, Daqing, Heilongjiang China

**Keywords:** Meta-analysis, Blonanserin, Risperidone, Schizophrenia

## Abstract

**Background:**

We conducted a systematic review and meta-analysis to evaluate the efficacy and safety of blonanserin and risperidone for the treatment of schizophrenia and to provide reliable pharmacotherapeutic evidence for in the clinical treatment of schizophrenia.

**Methods:**

We systematically searched the PubMed, Cochrane Library, Embase, Chinese Biomedical Literature Database (CBM), and China National Knowledge Infrastructure (CNKI) databases for head-to-head randomized controlled trials that compared blonanserin with risperidone for the treatment of schizophrenia. We extracted the following data: author, year, country, diagnostic criteria, sample size, course of treatment, dosage and outcomes. Our main endpoint was the changes in the Positive and Negative Syndrome Scale (PANSS) total scores. Meta-analysis of the included data was conducted by RevMan 5.3 software. We used the GRADE criteria to evaluate the certainty of the evidence.

**Results:**

A total of 411 studies were initially; 8 trials were eligible and were included in our analysis (N = 1386 participants). Regarding efficacy, there was no difference in changes in the PANSS total scores between the two groups (P > 0.05). In terms of safety, compared to risperidone, the incidence of serum prolactin increases and weight gain in the blonanserin group was lower (P<0.05), but the incidence of extrapyramidal symptoms (EPS) was higher (P<0.05).

**Conclusion:**

The efficacy of blonanserin is similar to that of risperidone, but it is unclear whether blonanserin is more effective than risperidone at improving cognitive and social function. More high-quality studies are needed to verify the efficacy and safety of blonanserin in the future.

**Supplementary Information:**

The online version contains supplementary material available at 10.1186/s12888-023-05240-7.

## Introduction

Schizophrenia is regarded as one of the most severe mental illnesses and may first present in adolescence [1]. According to a study of the global burden of disease, the number of patients with schizophrenia worldwide is approximately 20.9 million [2]. The clinical features of schizophrenia consist of positive and negative symptoms, social dysfunctions and cognitive impairments, which severely affect patients’ physical well-being, mental health and activities of daily living [3]. The Positive and Negative Syndrome Scale (PANSS) is commonly used to evaluate schizophrenia [4]. After accepting systematic therapy, most patients experience more than one recurrent schizophrenic episode. As a result, patients with schizophrenia typically require long-term treatment to maintain the treatment effect and decrease the rate of recurrence.

Antipsychotics, especially second-generation antipsychotics (SGAs), are recommended as the first-line therapy in the current treatment guidelines for schizophrenia [5]. Of these, risperidone is a representative medication that can effectively ameliorate both positive and negative symptoms in patients with schizophrenia. However, risperidone also has some side effects, including hyperprolactinemia [6]. To address these side effects, blonanserin has been gradually developed. Blonanserin is a relatively new SGA for the treatment of schizophrenia that has been approved in Japan, South Korea, and China [7]. It has a strong affinity for dopamine D_2_ and D_3_ receptors and 5-HT_2A_ receptors, which has a good curative effect on both the positive and negative symptoms of schizophrenia and may improve some cognitive symptoms and social function of patients [8–10]. But which is more effective? The conclusions are inconsistent. For example, some studies have shown that the efficacy of blonanserin is the same as that of risperidone [11, 12], whereas others have shown that the efficacy of blonanserin is superior [13]. In terms of safety, pharmacological evidence shows that blonanserin has a 94-fold higher affinity for D_2_ receptors than risperidone, which may lead to more extrapyramidal symptoms (EPS) and hyperprolactinemia [14]. However, in clinical practice, the conclusions have not been exactly the same. Thus, we performed a systematic review and meta-analysis to evaluate the efficacy and safety of blonanserin versus risperidone in the treatment of schizophrenia and to provide reliable evidence for the treatment of schizophrenia in clinical settings.

Previous meta-analyses have included trials that compared different medications with blonanserin [15–17], and some network meta-analysis included indirect comparisons of different drugs [18, 19], but these approaches might cause some biases. Any indirect comparisons may be subject to potential biases that are not present in a head-to-head direct comparison [20]. Therefore, we strictly included head-to-head studies. Previous studies also failed to investigate the improvement in cognitive and social function induced by blonanserin compared with other medications; thus, we also performed a qualitative analysis of the improvement in cognitive and social function induced by blonanserin and risperidone.

## Methods

### Protocol and registration

The meta-analysis was reported by the Preferred Reporting Items for Systematic Reviews and Meta-Analyses (PRISMA) statement [21]. The study protocol was registered at PROSPERO (CRD42022366600).

### Data sources and search strategy

The PubMed, Cochrane Library, Embase, Chinese Biomedical Literature Database (CBM), and China National Knowledge Infrastructure (CNKI) were searched for randomized controlled trials (RCTs) from inception to October 18, 2022. No language restrictions were applied. We used the following text and MeSH terms: “blonanserin” and “schizophrenia”. The complete search used for PubMed was: ((“Schizophrenia” [Mesh]) OR ((Schizophrenia*[Title/Abstract]) OR (Dementia Praecox [Title/Abstract]))) AND ((“blonanserin” [Supplementary Concept]) OR ((blonanserin [Title/Abstract]) OR (AD-5423[Title/Abstract]))). Table Supplement 1 shows the retrieval strategies for and retrieval results from PubMed.

### Literature inclusion criteria and exclusion criteria

The eligibility criteria were as follows: (1) Population: patients ≥ 18 years old with schizophrenia according to the Diagnostic Criteria for Research accompanying the International Statistical Classification of Diseases and Related Health Problems, 10th Revision for Mental and Behavioural Disorders (ICD-10) or the Diagnostic and Statistical Manual of Mental Disorders, Fourth Edition, Test Revision (DSM-IV-TR) diagnostic criteria. (2) Intervention: blonanserin with any dose, oral administration. (3) Comparison: risperidone with any dose, oral administration. (4) Outcome: The primary outcome was the changes in the PANSS total scores. The secondary outcomes included changes in the PANSS subscale scores; improvements in cognitive function and social function; and the incidence of any adverse events, EPS, serum prolactin increases and weight gain. The evaluation of adverse extrapyramidal events was based on scales, including the Drug Induced Extra Pyramidal Symptoms Scale (DIEPSS), the Barnes Akathisia Rating Scale (BARS), the Extrapyramidal Symptom Rating Scale (ESRS) and the Treatment Emergent Symptom Scale (TESS). The evaluation of increased serum prolactin levels was performed by laboratory examination, and hyperprolactinemia was defined as greater than 25 µg/L [22]. Weight gain was indicated by a ≥ 7% increase in the BMI compared with baseline data [23]. (5) Type of study: head-to-head RCTs of blonanserin and risperidone for the treatment of schizophrenia were included.

The exclusion criteria were as follows: (1) abstracts; (2) patients with severe liver and renal dysfunctions; (3) patients taking other antipsychotic medications at the same time, with the exception of lorazepam used to treat clinically significant agitation symptoms; and (4) patients with a history of drug allergy to blonanserin and risperidone.

### Study selection and data extraction

Study selection was performed by two reviewers (SW Deng and Q Xu) who independently screened the literature based on the eligibility and exclusion criteria. Duplicated and irrelevant articles were first excluded based on their titles and abstracts. Thereafter, the full texts of potentially eligible articles were downloaded and read to identify all eligible studies. Any disagreements were resolved by consulting the third reviewer (WL Jiang).

Data extraction was also performed independently by the two abovementioned reviewers (SW Deng and Q Xu). Any disagreements were resolved by consulting the third reviewer (WL Jiang). We extracted the following data from each selected study: author, year, country, diagnostic criteria, sample size, course of treatment, dosage and outcomes.

### Quality evaluation

Two quality evaluation methods were used to evaluate the literature. The Cochrane risk of bias tool [24] was used to evaluate the risk of bias in the included RCTs. The tool assessed the following seven domains: randomization sequence generation, allocation concealment, blinding of participants and personnel, blinding of outcome assessment, incomplete outcome data, selective reporting, and other bias. The modified Jadad scale [25] was used to evaluate the quality of RCTs. For the modified Jadad scale, there were four scoring items, with scale scores ranging from 0 to 7 points, with higher scores indicating greater quality. A total of 1–3 signified low quality, while 4–7 signified high quality [25]. Quality evaluation was also performed independently by the two reviewers (SW Deng and Q Xu). If there was any disagreement, the third reviewer (WL Jiang) was consulted to reach a consensus.

To assess the quality of the evidence on outcome indicators, we used the Grading of Recommendations, Assessment, Development and Evaluation (GRADE) [26], the criteria which included the risk of bias, inconsistency, indirectness, inaccuracy, and publication bias. The quality of evidence was classified as high, moderate, low, or very low.

### Statistical analysis

Categorical variables were expressed as relative risks (RRs) and 95% confidence intervals (95% CIs). Continuous variables are expressed as the mean standard deviation and 95% CI. Meta-analyses were performed using random effects models to account for clinical heterogeneity. Statistical heterogeneity between trials was assessed by the Cochrane Q test (P ≤ 0.1 indicated significance). All statistical analyses were performed using Review Manager (version 5.3).

## Results

### Study selection

**Fig. 1:**
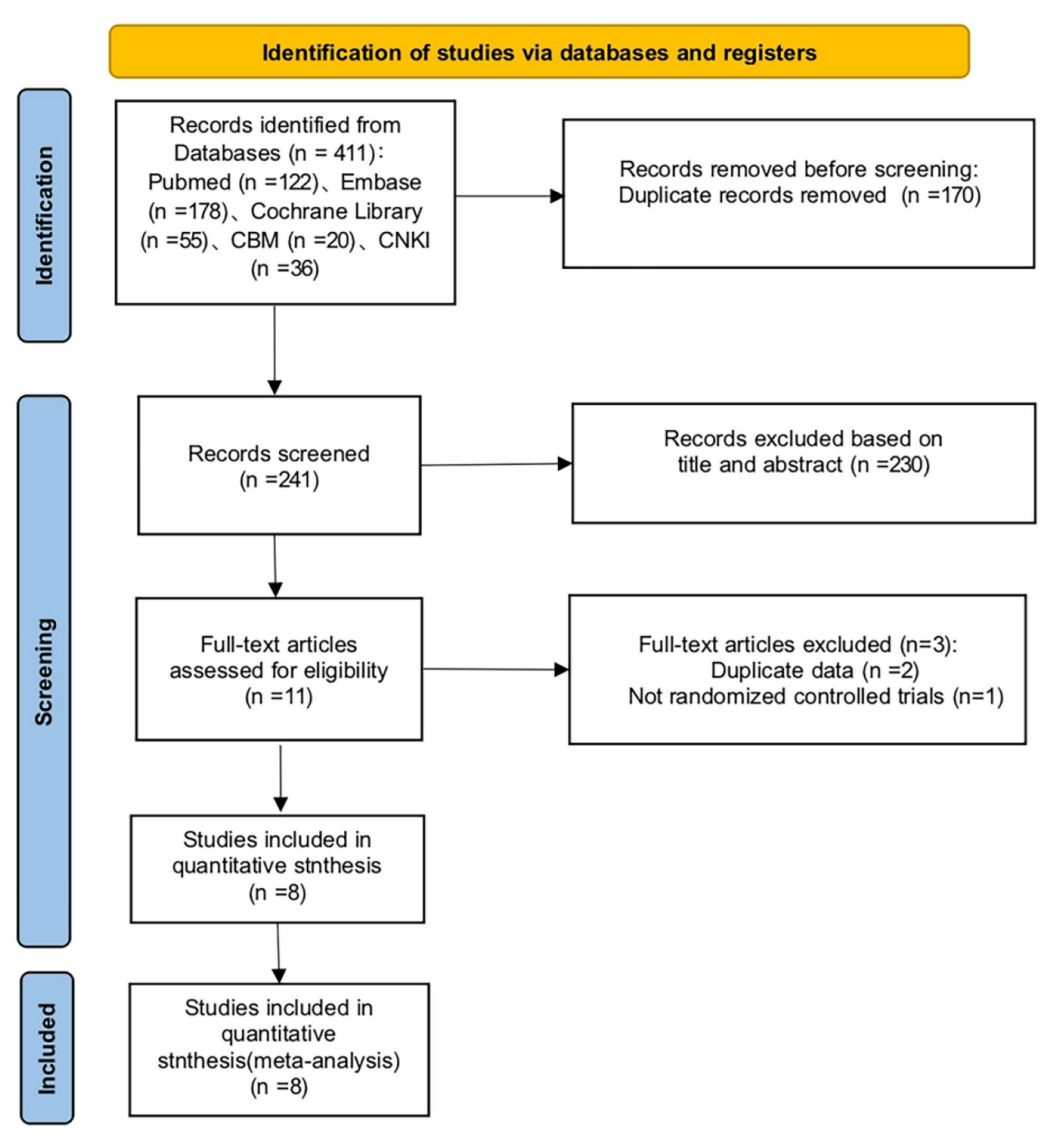
PRISMA flow diagram

A total of 411 records were found in the electronic databases. Following the removal of duplicates, 241 articles were screened for titles and abstracts. After reading the titles and abstracts, 230 unrelated articles were further excluded. Then, 11 articles were identified for full-text review. Of these, three articles were excluded: two articles were excluded because of duplicate data, and one article was excluded because it was not a randomized controlled trial. Ultimately, eight trials [11–13, 27–31] were included (Fig. 1).

### Study characteristics

These trials were published between 2010 and 2022 in four countries, namely, the United States of America, China, Japan, and Korea. The sample size of the individual trials ranged from 73 to 301. All trials reported efficacy and safety. The characteristics of the included trials are presented in Table 1.

**Table 1:** Characteristics of the included trials

Trial	Country	diagnostic criteria	Sample size (blonanserin/risperidone)	Course of treatment	Dosage	Outcomes
Gou YH 2022	China	ICD-10	100(50/50)	8 weeks	blonanserin: 8–16 mg/d risperidone: 2–6 mg/d	PANSS
Harvey PD 2020	USA	ICD-10	301(156/145)	8 weeks	blonanserin: 8–24 mg/d risperidone: 2–6 mg/d	PANSS, PANSS-P, PANSS-N, PANSS-G
Li H 2015	China	DSM-IV-TR	264(130/134)	8 weeks	blonanserin: 8–24 mg/d risperidone: 2–6 mg/d	PANSS, PANSS-P, PANSS-N, PANSS-G
Liu Q 2016	China	ICD-10	298(148/150)	8 weeks	blonanserin: 8–24 mg/d risperidone: 2–6 mg/d	PANSS, PANSS-P, PANSS-N, PANSS-G, PSP
Sun L 2022	China	ICD-10	92(46/46)	8 weeks	blonanserin: 8–24 mg/d risperidone: 2–6 mg/d	PANSS, PANSS-P, PANSS-N, PANSS-G, MCCB
Wang S 2019	China	ICD-10	75(37/38)	8 weeks	blonanserin: 8–24 mg/d risperidone: 2–6 mg/d	PANSS, SDS
Yang J 2010	Korea	ICD-10	183(92/91)	8 weeks	blonanserin: 8–24 mg/d risperidone: 2–6 mg/d	PANSS, PANSS-P, PANSS-N, PANSS-G
Zhang HW 2021	China	ICD-10	73(36/37)	12 weeks	blonanserin: 8–24 mg/d risperidone: 2–6 mg/d	PANSS, PANSS-P, PANSS-N, PANSS-G, MCCB, PSP

**Abbreviations: ICD-10**: the Diagnostic Criteria for Research accompanying the International Statistical Classification of Diseases and Related Health Problems, 10th Revision for Mental and Behavioral Disorders; **DSM-IV-TR**: the Diagnostic and Statistical Manual of Mental Disorders, Fourth Edition, Test Revision; **PANSS**: Positive and Negative Syndrome Scale; **PANSS-P**: PANSS-Positive subscale; **PANSS-N**: PANSS-Negative subscale; **PANSS-G**: PANSS-General psychopathology subscale;**PSP**: the Personal and Social Performance Scale; **MCCB**: The MATRICS Consensus Cognitive Battery; **SDS**: Sheehan disability scale

### Study quality

**Fig. 2:**
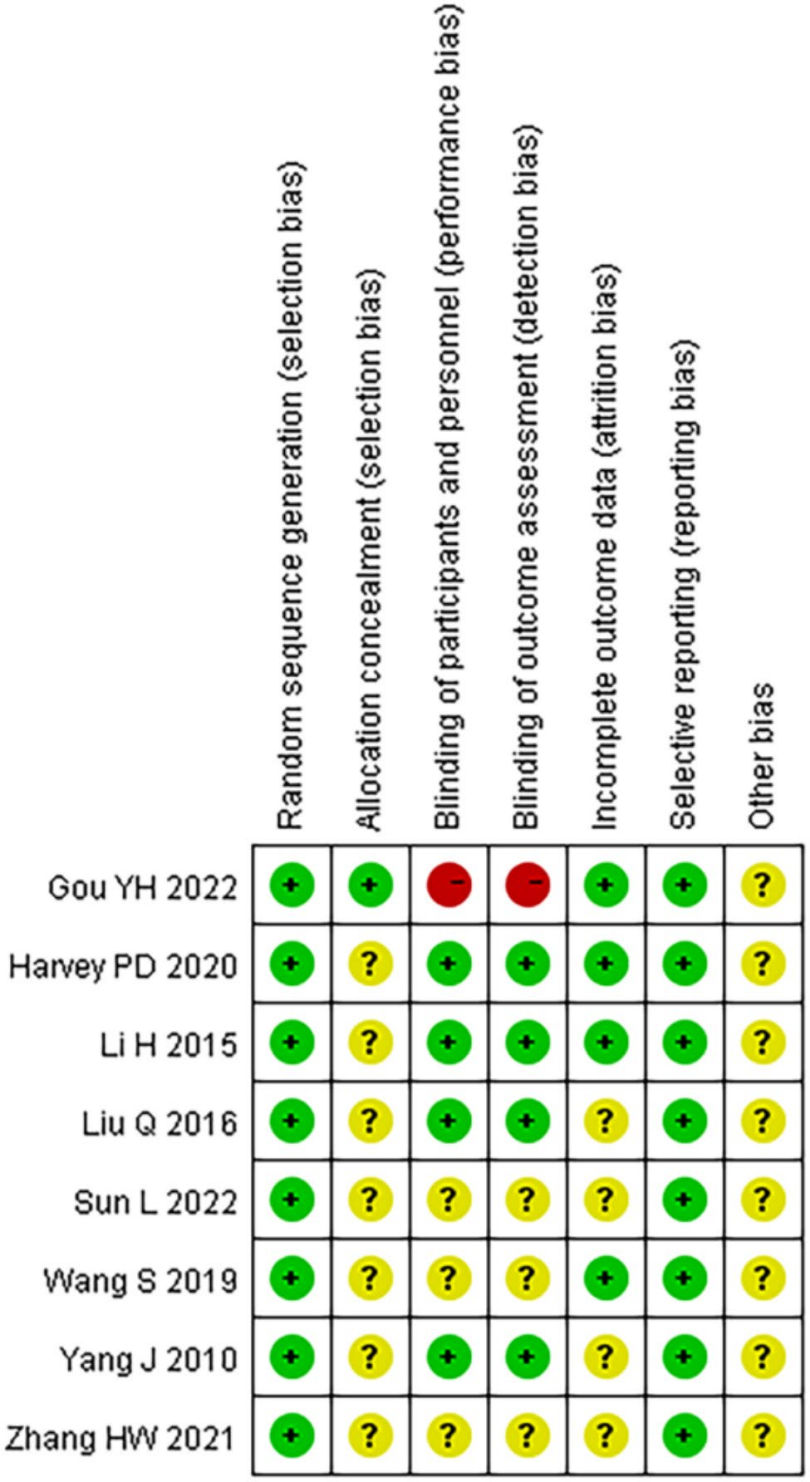
Risk-of-bias summary

The details of the risk-of-bias assessment for each included trial are summarized in Fig. 2. All eight RCTs reported the baseline situation of patients. All eight studies described the generation of random sequences: four studies were double-blinded, and one study performed allocation concealment. According to the Jadad scale, four studies had a score of four, three studies had a score of five, and one study had a score of six. Therefore, the scores of all included studies were greater than or equal to four, which indicates that the quality of the included studies was relatively high.

### Meta-analysis of PANSS total scores

**Fig. 3:**
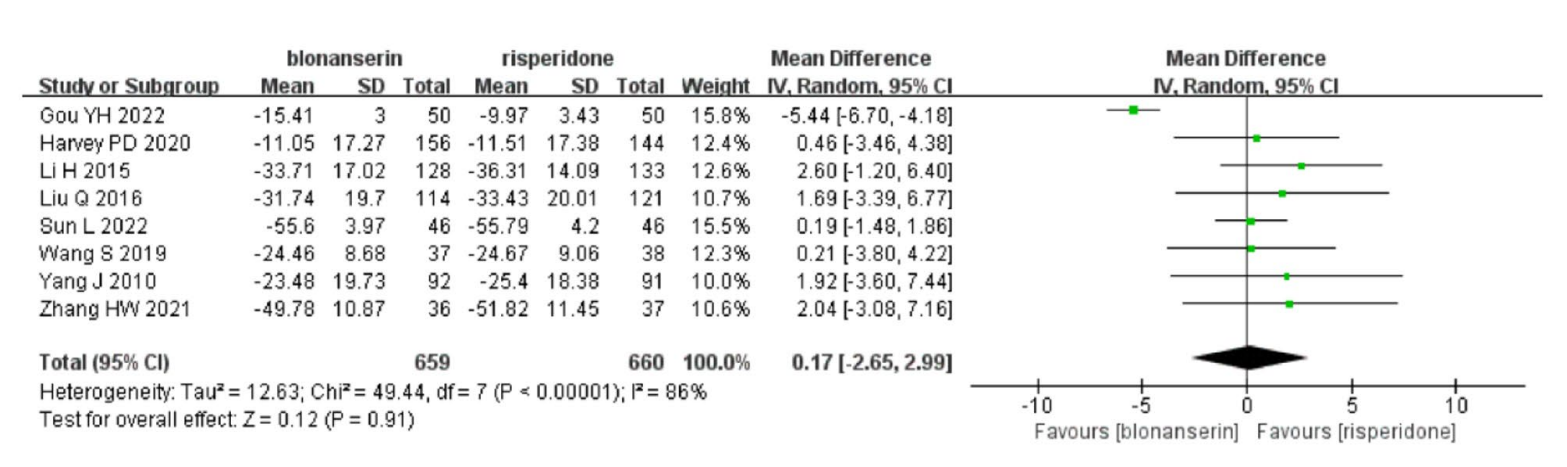
Forest plot for PANSS total scores

The primary outcome was reported in eight trials [11–13, 27–31] with a total of 1319 participants. A pooled analysis of the eight trials showed that there was no difference between the blonanserin group and the risperidone group (MD = 0.17, 95% CI: -2.65–2.99, I^2^ = 86%, P = 0.91; Fig. 3). We removed each article one at a time to perform sensitivity analysis, and the range of results was P = 0.23 to 1.00. See Table Supplement 2 for details.

### Meta-analysis of PANSS subscale scores

Five trials [11, 12, 27, 28, 31] with 858 participants provided data regarding the PANSS subscale scores. For PANSS-Positive subscale scores and PANSS-Negative subscale scores, there were no differences between the blonanserin and risperidone groups (P > 0.05). However, for the PANSS-General psychopathology subscale scores, greater improvement was observed in the risperidone group (P<0.05; Table 2).

**Table 2:** Meta-analysis results of PANSS subscale scores

Outcome	Mean Difference	95% CI	I^2^	P
PANSS-Positive subscale scores	0.06	-0.53–0.66	0%	0.83
PANSS-Negative subscale scores	-0.28	-0.85–0.28	0%	0.33
PANSS-General psychopathology subscale scores	1.20	0.28–2.12	0%	0.01

### Qualitative analysis of cognitive and social function

Among the eight included studies, two studies [12, 31] compared the effect on social function evaluated by the MATRICS Consensus Cognitive Battery (MCCB) [32]. One study demonstrated superior improvement in the blonanserin group compared with the risperidone group [12]. However, Zhang [31] found that there was no significant difference in the overall cognitive ability of patients with schizophrenia between the blonanserin and risperidone groups.

Of the eight included studies, three articles [29–31] compared the effect on social function evaluated by the Sheehan Disability Scale (SDS) or the Personal and Social Performance Scale (PSP). Of these, two showed superior improvement in the blonanserin group compared with the risperidone group [30, 31]. However, inconsistent outcomes were found in another study [29].

### Meta-analysis of adverse events

#### Any adverse events

**Fig. 4:**
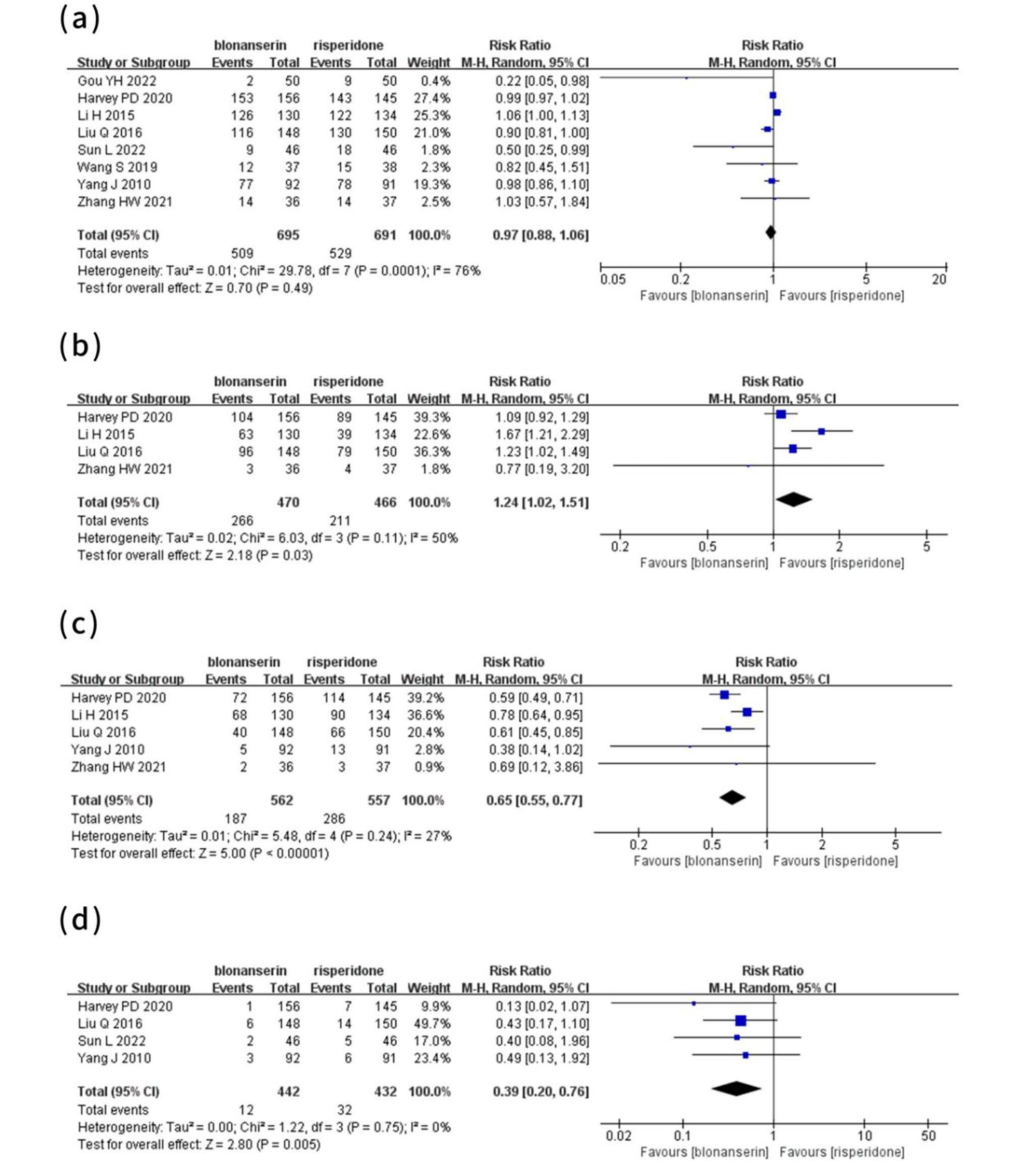
Forest plot for adverse events

Eight trials [11–13, 27–31] involving 1386 participants all reported adverse events. Among them, the incidence of adverse events reported in four trials was relatively high, but most of them were mild. Pooled analysis of the eight trials demonstrated that there was no difference in any adverse events between the blonanserin and risperidone groups (P > 0.05; Fig. 4-a).

#### Extrapyramidal adverse events

The risk of EPS was assessed in four studies [11, 27, 29, 31] with a total of 936 participants. The meta-analysis of the four studies indicated that compared with blonanserin, the incidence of EPS was lower in the risperidone group (P<0.05; Fig. 4-b).

#### Increase of serum prolactin

In five trials [11, 27–29, 31] involving 1119 participants, an increase in serum prolactin was reported. The pooled results showed that compared to risperidone, the incidence of serum prolactin increases was lower in the blonanserin group (P<0.05; Fig. 4-c).

#### Weight gain

The risk of weight gain was assessed in four trials [12, 27–29] involving 874 participants. The meta-analysis of the four studies indicated that compared with risperidone, the incidence of weight gain was lower in the blonanserin group (P<0.05; Fig. 4-d).

### GRADE certainty of evidence

**Fig. 5:**
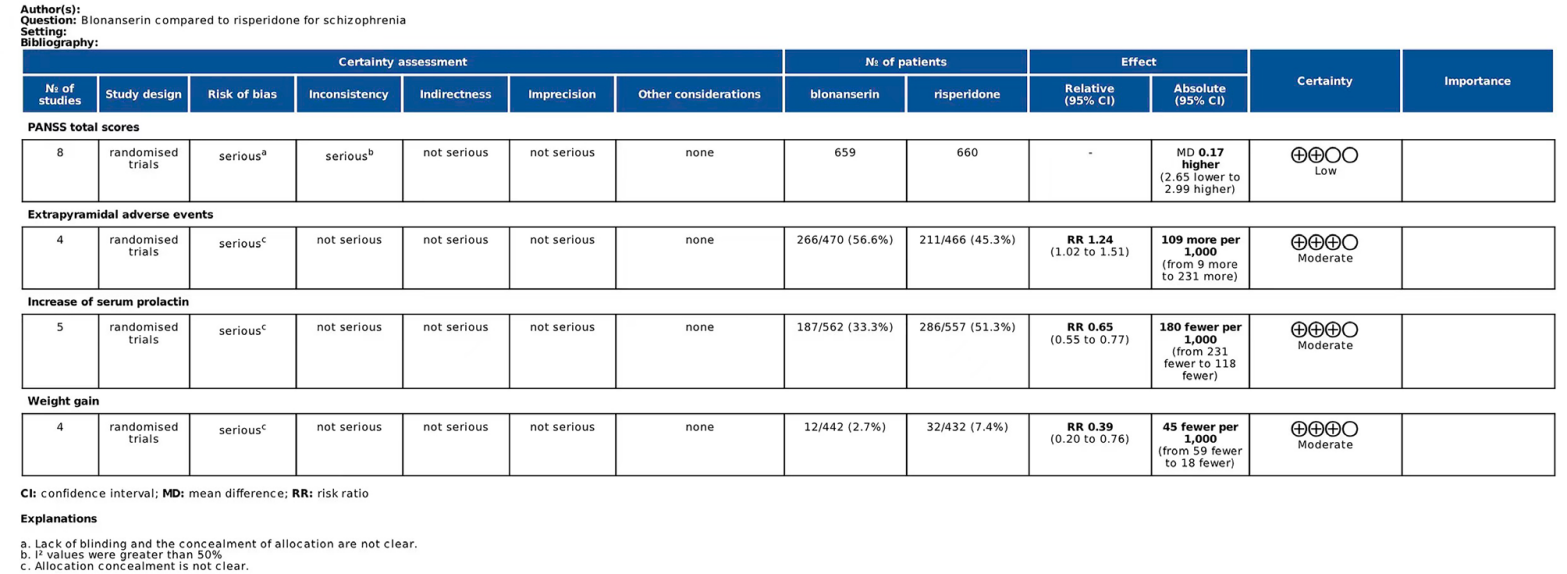
Quality of evidence

GRADE evidence profiles for the PANSS total scores and adverse reactions are shown in Fig. 5. According to the criteria of the GRADE approach, the quality of the evidence of the PANSS total scores was rated as low. The quality of evidence was moderate for EPS, serum prolactin increases and weight gain.

## Discussion

### Main findings

Our meta-analysis comprehensively and systematically reviewed the current head-to-head RCTs and compared the efficacy and safety of blonanserin with risperidone for treating schizophrenia. Although our results showed that the difference in efficacy was not significant, there were some differences in adverse reactions, which were similar to the results of some previously published meta-analyses [15, 18, 19].

For efficacy, we found that there was no statistically significant difference in PANSS total scores, positive symptoms and negative symptoms between the two groups, indicating that the efficacy of blonanserin was equivalent to that of risperidone, and both of them could effectively improve the mental symptoms of patients with schizophrenia. However, we found that compared with blonanserin, greater improvements were observed in the PANSS-General psychopathology subscale scores in the risperidone group. When we changed the effect size to SMD, we found that the effect size was very small (SMD<0.2), which is not clinically significant. In terms of safety, compared with risperidone, the incidence of serum prolactin increases and weight gain is lower in the blonanserin group, but the incidence of EPS is higher. EPS, hyperprolactinemia and weight gain are detrimental to patients’ health and can lead to adverse effects, such as endocrine disorders, disease burden increase, and poor compliance with drugs [33, 34]. Thus, we should not only consider the efficacy but also pay attention to the safety to prevent these adverse reactions. EPS is a common adverse reactions to blonanserin, but they often have a mild severity [35]. It is possible that the occurrence of EPS is related to the blood concentration of blonanserin [36]. The specific mechanism of weight gain caused by antipsychotic drugs is unclear [33]. This may be associated with decreased activity due to gene and drug-induced sedation and an increase in appetite as a result of receptors such as 5-HT_2C_ and H_1_ [37–39].

Cognitive impairment is one of the core symptoms of schizophrenia, resulting in significant impairment of social function and a decline in quality of life. Currently, there is no effective treatment for cognitive symptoms. Blonanserin has been shown to have a high affinity for the D_3_ receptor, suggesting that it may improve cognitive symptoms [40]. Animal studies also showed that blonanserin could block the dopamine D_3_ receptor, promote the release of dopamine and acetylcholine in the cortex, and improve cognitive impairment and social function [41–43]. These research results all suggest that blonanserin may improve some cognitive and social functions in patients with schizophrenia. However, through qualitative analysis, we are unable to determine whether blonanserin is superior to risperidone in improving cognitive and social functions because the number of trials is limited and assessments of cognitive and social functions are inconsistent among trials. Therefore, it remains to be confirmed further in large-sample randomized controlled trials.

In summary, our meta-analysis further confirmed that blonanserin is an effective and safe medication for the treatment of schizophrenia and that the efficacy of blonanserin is similar to that of risperidone.

### Implication for clinical practice

It has been shown that blonanserin may be beneficial in patients with treatment-resistant schizophrenia and dopamine supersensitivity psychosis [44]. Additionally, blonanserin may be a safe treatment option for adolescent schizophrenia that can be used seamlessly from adolescence to adulthood [45, 46]. In our meta-analysis, the results suggested that blonanserin may be as effective as risperidone for the treatment of schizophrenia. However, one important thing to note is that the incidence of EPS was lower in the risperidone group than in the blonanserin group. Although EPS is mild or moderate and can be eliminated by using antagonists, blonanserin should still be used cautiously [33]. To ameliorate EPS, the dose can be adjusted by measuring the blood concentration. At present, there is research to find another possible way to reduce EPS by converting a blonanserin tablet/powder to a transdermal patch [47]. This approach should be further examined in future research. The increase in prolactin caused by antipsychotic drugs may lead to irregular menstruation in women and sexual dysfunction in men, affecting patient compliance [33]. A study found that blonanserin may be a better choice for young and middle-aged female patients with schizophrenia because it is well tolerated and has a low tendency to cause metabolic side effects and hyperprolactinemia [48]. In our study, we found that the risk of prolactin increases in blonanserin was lower than that in risperidone, so women of childbearing age could choose drugs with less effect on prolactin, such as blonanserin, for more prudent treatment. In addition, we need to monitor the prolactin level of patients during the treatment. Similarly, in our study, we found that the risk of weight gain associated with blonanserin was lower than that associated with risperidone. Therefore, for obese patients, we can choose drugs that have less impact on weight, such as blonanserin.

### Strengths and limitations

On the one hand, the advantage of our meta-analysis is that it explores safety and efficacy in a larger population. We included different races, and the evidence obtained from a larger sample size may be more reliable. Kishi et al. conducted two meta-analyses, but only two articles were included [15, 16]. Furthermore, our research also has some differences from previous studies in methodology. Some influential factors have been considered, and our method is more rigorous. Kishi et al. used risperidone and paliperidone as the same control, blonanserin was used as an intervention, and its sample was also small [17]. Our study, however, used only risperidone as a control. Kishi et al. included randomized trials of Japanese patients only, and most of the randomized trials permitted the use of additional anxiolytics, sleeping pills or additional antipsychotics during the study [18]. In contrast, the randomized trials included in our study were not permitted to take other antipsychotics concurrently, and only lorazepam was allowed to treat symptoms of clinically significant agitation. We thought these factors might have an impact on the results. On the other hand, in comparison to the previous meta-analysis, we not only performed a meta-analysis of the efficacy and safety of blonanserin and risperidone in the treatment of schizophrenia but also performed a qualitative analysis on the improvement of cognitive function and social function of blonanserin and risperidone. Additionally, we used the GRADE approach to assess the certainty of the evidence.

Of course, there are also some limitations to our meta-analysis that may affect the interpretation of the results. First, it is difficult to rule out the existence of publication bias since only eight trials were included in our meta-analysis. Second, each of the studies we included used different cognitive and social function scales. Third, due to the limitation of language, we could not retrieve the relevant data from the Japanese literature. Therefore, more studies on the treatment of schizophrenia with blonanserin and risperidone are required to further verify their efficacy and safety.

## Conclusions

The efficacy of blonanserin is similar to that of risperidone. Risperidone is associated with a lower incidence of EPS, while blonanserin is associated with lower incidences of serum prolactin increases and weight gain. However, it is unclear whether blonanserin is superior to risperidone in improving cognitive and social function. More studies are required to clarify which medication is more effective, safer, and more prone to patient compliance in the treatment of schizophrenia to provide an evidence-based reference for the rational clinical application of antipsychotic drugs and safe treatment.

### Electronic supplementary material

Below is the link to the electronic supplementary material.

**Supplementary Material 1**.

## Data Availability

The datasets used and/or analysed during the current study are available from the corresponding author on reasonable request.
