# Supplementary material for: Efficacy and safety of blonanserin versus risperidone in the treatment of schizophrenia: a systematic review and meta-analysis of randomized controlled trials
Source: BMC Psychiatry. 2023 Oct 11;23:740. doi: 10.1186/s12888-023-05240-7 (PMC10568781; doi:10.1186/s12888-023-05240-7)
Supplement: Supplementary file 1 — Supplementary Material 1. [file 12888_2023_5240_MOESM1_ESM.docx]

**Table Supplement1:** The retrieval strategies and retrieval results of PubMed

| No. | Content | Result |
| --- | --- | --- |
| #1 | Search: "Schizophrenia"[Mesh] Sort by: Most Recent | 113,055 |
| #2 | Search: (Schizophreni*[Title/Abstract]) OR (Dementia Praecox[Title/Abstract]) Sort by: Most Recent | 139,752 |
| #3 | Search: ("Schizophrenia"[Mesh]) OR ((Schizophreni*[Title/Abstract]) OR (Dementia Praecox[Title/Abstract])) Sort by: Most Recent | 159,987 |
| #4 | Search: "blonanserin" [Supplementary Concept] Sort by: Most Recent | 112 |
| #5 | Search: (blonanserin[Title/Abstract]) OR (AD-5423[Title/Abstract]) Sort by: Most Recent | 179 |
| #6 | Search: ("blonanserin" [Supplementary Concept]) OR ((blonanserin[Title/Abstract]) OR (AD-5423[Title/Abstract])) Sort by: Most Recent | 181 |
| #7 | Search: (("Schizophrenia"[Mesh]) OR ((Schizophreni*[Title/Abstract]) OR (Dementia Praecox[Title/Abstract]))) AND (("blonanserin" [Supplementary Concept]) OR ((blonanserin[Title/Abstract]) OR (AD-5423[Title/Abstract]))) Sort by: Most Recent | 122 |

**Table Supplement2** Results of sensitivity analysis

| Study omitted | MD | 95%CI | P |
| --- | --- | --- | --- |
| Gou YH (2022) | 0.75 | -0.48, 1.98 | 0.23 |
| Harvey PD (2020) | 0.15 | -2.96, 3.25 | 0.93 |
| Li H (2015) | -0.19 | -3.17, 2.78 | 0.90 |
| Liu Q (2016) | -0.01 | -3.02, 3.01 | 1.00 |
| Sun L (2022) | 0.24 | -3.19, 3.67 | 0.89 |
| Wang S (2019) | 0.19 | -2.92, 3.30 | 0.91 |
| Yang J (2010) | -0.02 | -3.02, 2.97 | 0.99 |
| Zhang HW (2021) | -0.05 | -3.05, 2.95 | 0.97 |
